# UV Light Irradiation Effects in P-Doped Diamonds: Total Content Determination of Phosphorus Donors

**DOI:** 10.3390/ma15249048

**Published:** 2022-12-18

**Authors:** Kirill Boldyrev, Sergey Klimin, Viktor Denisov, Sergey Tarelkin, Mikhail Kuznetsov, Sergey Terentiev, Vladimir Blank

**Affiliations:** 1 Institute of Spectroscopy, Russian Academy of Sciences, 108840 Moscow, Russia; 2 Technological Institute for Superhard and Novel Carbon Materials, 108840 Moscow, Russia; 3 The All-Russian Research Institute for Optical and Physical Measurements, 119361 Moscow, Russia

**Keywords:** phosphorus-doped diamond, total concentration, FTIR

## Abstract

Upon the UV light irradiation of single-crystal diamonds doped with phosphorus, several effects have been observed. The integral intensity of phosphorus lines in FTIR absorption spectra under UV radiation was increased. A saturation effect depending on the power of the laser radiation was demonstrated. Narrowing of the phosphorus lines, as well as the redistribution of the intensities in their doublets caused by the Jahn–Teller distortion of the donor ground state, was observed. It was found that these effects are associated with the decompensation of the phosphorus donors. An easy, fast, sensitive, and nondestructive, fully optical method for the determination of the total phosphorus donor’s concentration in semiconducting diamonds, as well as its compensation ratio, was proposed.

## 1. Introduction

Currently, there is a need to create new materials for radiation-resistant, high-frequency, and high-power electronics, as well as for quantum information storage devices and deep ultraviolet (DUV) optoelectronics [1,2]. Diamond has several advantages, such as a wide band-gap (5.47 eV), high breakdown electric field (>10 MV/cm), high hole and electron mobilities (>2000 cm^2^/(V × s)), and high thermal conductivity (22 W/(cm × K)). For decades, diamond was considered to be a typical insulator due to the difficulties in doping it caused by electroactive impurities to control its conductivity. However, with the development of synthetic growth methods, such as the high-pressure high-temperature (HPHT) gradient one [3,4,5,6,7,8,9] and chemical vapor deposition (CVD) [10,11,12,13,14,15,16,17,18,19,20,21], it became possible to manage the content of impurities and the electrical properties of diamonds. 

Boron-doped p-type diamonds are fairly easy to produce [3,4,5,6,10,11,12]. However, for solid state physics and for the development of quantum computers, n-type diamonds doped with phosphorus are demanded [7,8,9,13,14,15,16,17,18,19,20,21]. The boron (B) acceptor level of 0.37 eV above the valence band maximum and the phosphorus (P) donor level of 0.6 eV below the conduction band minimum are formed with the substitutional B or P incorporation into the diamond lattice. These B acceptor and P donor levels are relatively deep, with only ∼0.2% of acceptor holes and ∼0.001% of donor electrons being ionized at room temperature, limiting the operation of diamond-based electronic devices [22,23,24,25]. High phosphorus doping of diamond above ~10^20^ cm^−3^ reduces the activation energy by up to 0.027 or 0.05 eV [23,26].

Recently, the energy diagrams of the electronic levels of the even boron acceptor states and odd phosphorus donor states were studied in detail by electronic Raman scattering as well as by Fourier Transform Infra-Red (FTIR) absorption, respectively [6,9]. The energy-level diagram of boron acceptor states shows the 2-meV spin–orbit splitting of the 1s(A_1_) ground acceptor state into the 1*s*(*p*_3_*_/_*_2_) and 1*s*(*p*_1_*_/_*_2_) states and the *ns* (*n* = 2, 3, 4) exited acceptor states with their 4, 6, 8-meV spin–orbit splitting, correspondingly. The 1*s* → *ns* electronic transitions reveal the fine structure, consisting of three groups with four bands for the 1*s* → 2,3*s* transitions and two bands for the 1*s* → 4*s* transition [6]. In accordance with the FTIR absorption spectra, the transitions 1*s* → *np*_±_ and 1*s* → *np*_0_ (*n* = 2, 3, 4) are identified. The doublet structure of the electronic transitions 1*s*(B_2_, E) → 2*p*_±_, 1*s*(B_2_, E) → 4*p*_0_, 1*s*(B_2_, E) → 3*p*_±_ and 1*s*(B_2_, E) → 4*p*_±_ with a splitting of 1.05 meV was observed [9]. The reason for these doublet structures is the dynamic Jahn–Teller effect, which splits the energy level of the 1*s*(T_2_) state in the T*_d_* symmetry into those of the 1*s*(B_2_) ground state and 1*s*(E) state in the D_2*d*_ symmetry. Notably, the 1s(A_1_) ground boron acceptor state is singlet, while the 1*s*(T_2_) ground phosphorus donor state is triplet.

One of the most important problems in the creation of electronic devices based on doped semiconducting diamonds is the determination of the total concentration of donors (N_D_) or acceptors (N_A_) and their spatial distribution in samples. Electrical methods (Hall effect study) show only the net concentration (N_D_-N_A_) of uncompensated charges and degree of compensation N_A_/N_D_. For these purposes, Hall measurements should be performed in a wide temperature range (up to full ionization of donors—at least 1000 °C for P donors), requiring the formation of good ohmic contacts and a high-temperature vacuum chamber with electrical ports [18,24]. Secondary Ion Mass Spectrometry (SIMS) analysis is difficult to perform. Moreover, it only provides information about the total concentration of impurities in diamond, but not about the crystallographic positions of impurities in the diamond lattice. The SIMS technique is a destructive method and leaves surface craters of 150 × 150-μm^2^ and approximately 10 μm in depth. Moreover, diamond samples are small and should be placed in a large, flat, conductive matrix to perform measurements. For 10% accuracy of SIMS measurements, implanted standards are needed. The FTIR absorption optical method was proposed for the determination of the net (N_D_-N_A_) concentration of phosphorus donors or the net (N_A_-N_D_) concentration of boron acceptors in diamonds [24]. However, in the presented version (without active exposure to light), it did not take into an account the compensated impurities, and it is only suitable for the determination of the net-doping concentration. The cathodoluminescence spectroscopy measures the total dopant density, N_D_ for n-type diamond or N_A_ for p-type diamond [27]. 

In this paper, we report an increase in the intensity and a narrowing of the phosphorus lines in the IR absorption spectra of diamond samples doped by phosphorus upon their irradiation by a UV laser. The impact of the laser radiation power has also been investigated. On the basis of these observations, a method for the quantitative determination of the total phosphorus concentration in diamond, as well as of the compensation ratio, has been proposed. The n-type conductivity of phosphorus-doped diamond at room temperature can be highly limited due to the problem of compensation rather than by the donor concentration; therefore, measuring the ratio of compensated donors is important.

## 2. Materials and Methods

### 2.1. Sample Preparation and Characterization

**Figure 1:**
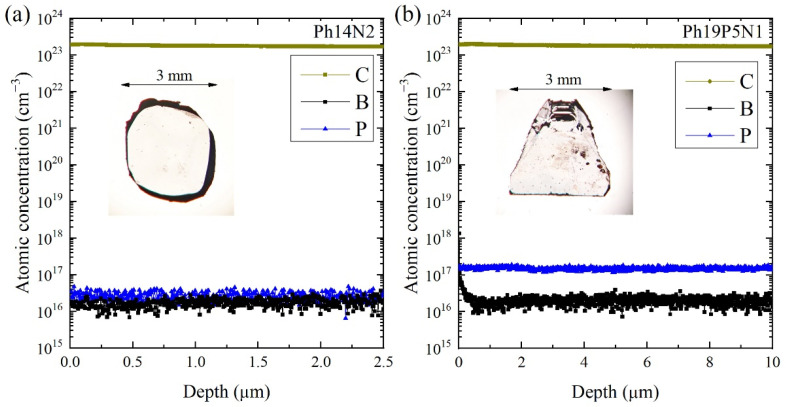
SIMS analysis for the content of boron and phosphorus of samples (**a**) Ph14N2 and (**b**) Ph19P5N1. The insets show photographs of the corresponding crystals.

Phosphorus-doped diamond single crystals Ph14N2 and Ph19P5N1 were grown by the temperature gradient method under high pressure at 5.5 GPa and a high temperature at 1440 °C in the “toroid”-type high-pressure apparatus. Fe-Al-C alloy (91:5:4 by wt. %) was used as the solvent metal. Aluminum was added to the solvent as the nitrogen getter. High-purity (99.9995%) graphite was used as the carbon source. Amorphous phosphorus powder was added to the carbon source in two concentrations as the doping agent. Growth mixture was supplied with 2% and 10% atomic percent of amorphous phosphorus with respect to carbon amount. Synthetic diamond crystals with a (100) surface and approximately 0.5–0.6 mm across were used as a seed material. The temperature in the reaction cell during the growth run was directly measured by a Pt6%Rh–Pt30%Rh thermocouple with accuracy ±2 °C. Two diamond plates with an edge of ∼3 mm and thickness of 250 microns were cut with a laser from grown diamond crystals and then mechanically polished. In this work, we studied two P-doped single crystal diamond plates with concentrations of ∼2 × 10^16^ cm^−3^ (Ph14N2, see insert in Figure 1a) and ∼1.1 × 10^17^ cm^−3^ (Ph19P5N1, see insert in Figure 1b), measured by SIMS with the uncertainty of 10%. The samples also contained trace concentrations of boron (∼1 × 10^16^ cm^−3^), which entered uncontrollably from the growth environment (see Figure 1).

The crystalline perfection of P-doped single crystal diamond plates (Ph14N2 and Ph19P5N1) was characterized by Raman spectroscopy. Raman spectra of the P-doped diamond plates were obtained using a homemade multichannel triple-stage spectrometer (a 0.5-m focal length double subtractive monochromator and a 1.2-m focal length spectrometer) with a 0.6 cm^−1^ spectral resolution. The 1332.5-cm^−1^ Raman peaks with the full width at half maximum (FWHM) of ~1.8 cm^−1^ were almost the same as that observed from the unstressed IIa-type single-crystal diamond and indicated the absence of strain in P-doped diamond plates. No defect-induced bands in the Raman spectra of our diamond plates with low phosphorus doping were observed in comparison with those of the highly P-doped films and the highly B-doped diamonds [20,28].

### 2.2. Spectroscopic Measurements

The infrared (IR) absorption spectra of P-doped diamond single crystals were measured with a high-resolution Bruker IFS 125 HR vacuum Fourier transform spectrometer in the 1800 to 7000 cm^−1^ spectral region with a spectral resolution up to 0.2 cm^−1^ at temperatures between 10 and 300 K using a closed-circle helium cryostat Cryomech PT-403. A tungsten–halogen light source and an InSb detector were used for the given spectral region. A laser beam with the same diameter as the sample aperture (~2.5 mm in diameter) passed into the sample compartment through the CaF_2_ window. A laser beam falling at an angle 15° to the main optical axis of the Fourier spectrometer was applied. A continuous-wave 266-nm Nd:YAG fourth-harmonic laser with maximum power of 100 mW was used. The laser power was controlled using a Spectra-Physics 1918-C laser power meter.

## 3. Results and Discussion

### 3.1. Study of IR Spectra under Laser Irradiation

**Figure 2:**
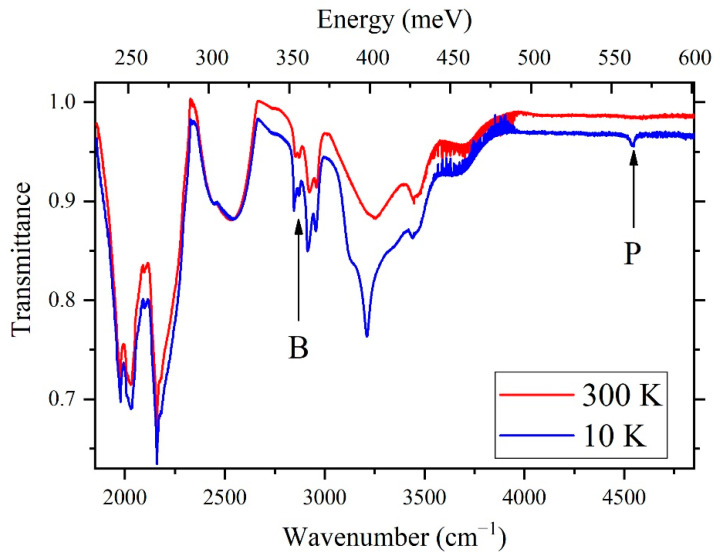
Transmission spectra of diamond sample Ph14N2 in the IR region (1800–4800 cm^−1^) at 10 (blue) and 300 K (red). The spectra are shifted for convenience.

The transmission spectra for the Ph14N2 diamond plate at room and low temperature are shown (Figure 2). Narrow lines appeared upon cooling, which corresponds to the well-known absorption transitions in phosphorus at ~4500 cm^−1^ (~560 meV) [9] and in boron at ∼2800 cm^−1^ (370 meV) [4].

**Figure 3:**
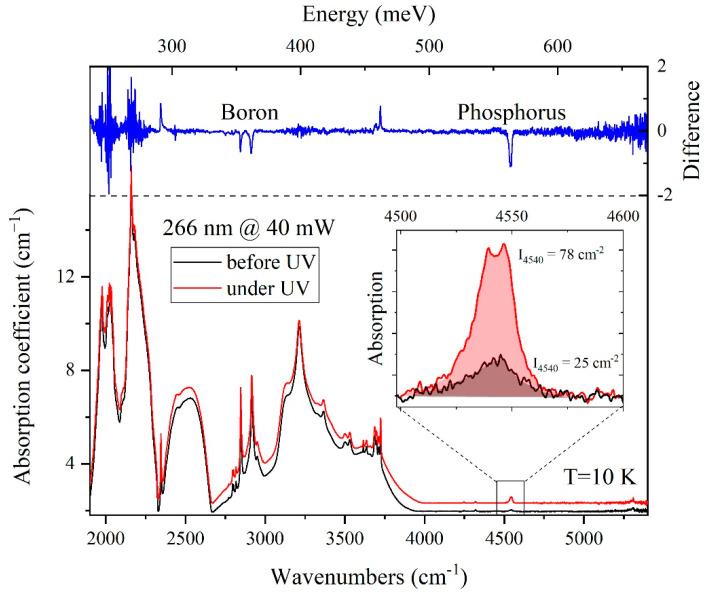
Absorption spectra of diamond Ph14N2 before (black) and during (red) laser irradiation with λ = 266 nm and power 40 mW; blue spectrum—subtraction of red spectrum from black one. The spectra are shifted for convenience.

For the Ph14N2 sample, a series of transmission spectra were measured before, during and after laser irradiation with *λ* = 266 nm (4.66 eV) and a power 40 mW (Figure 3) at a temperature of 10 K. It can be seen that under irradiation, the intensity of the phosphorus line at 4540 cm^−1^ (the most intensive electronic transition 1*s*(B_2_, E) → 2p_±_) undergoes a threefold increase (integral intensity is 78 vs. 25 cm^−2^ with and without illumination, respectively). In addition, an increase in the absorption line intensities associated with boron is observed. This directly indicates the effect of the decompensation of donor–acceptor pairs |N_D_-N_A_|. After the termination of irradiation, the spectrum returns to its original state.

**Figure 4:**
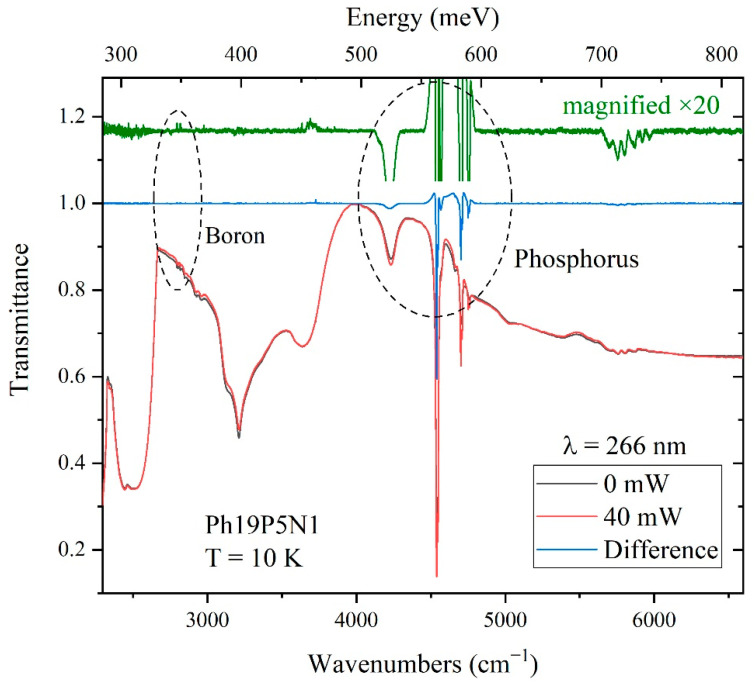
Transmission spectra of diamond sample Ph19P5N1 at 10 K without (black) and with (red) irradiation by laser; black–red difference spectrum (blue) and magnified difference spectrum (green). Irradiation by laser with wavelength *λ* = 266 nm and power 40 mW.

For the Ph19P5N1 sample with a higher concentration of phosphorus, IR transmission spectra were measured at 10 K without and with irradiation by a UV laser with *λ* = 266 nm and power 40 mW (see Figure 4). Increased intensity of the phosphorus line was observed, while no changes were visible in the region of boron transitions. While this increase was not significant in terms of the absolute value, it appeared more dramatic compared to sample Ph14N2: the difference line in the phosphorus range had a biconvex shape. This was due to the different spectral widths of the phosphorus absorption lines in the spectra of the sample without and with the influence of radiation. Interestingly, the spectral lines narrowed under irradiation.

The observed increase in the intensity of the absorption lines of both samples upon the UV laser irradiation of phosphorus-doped diamonds can be explained by the generation of excess free charges, which can transfer compensated phosphorus to a neutral state. The presence of these absorption lines can be explained by the electron transitions from the ground to excited states of neutral phosphorus impurities [9]. Since only the neutral centers can absorb light and the compensated impurities remain ionized, the resulting spectral features appear only due to the net impurities |N_D_-N_A_| [24]. Since the compensated impurities do not participate in IR optical absorption, it has generally been accepted that IR absorption could not be used for total impurity determinations. Our study shows that exposure to UV irradiation makes it possible to change the states of compensated phosphorus centers and make them optically active. A similar situation was observed for silicon samples doped by phosphorus and boron upon irradiation with light [29,30,31,32]. In this case, pairs of electrons and holes are generated, which also fill unpopulated donor and acceptor levels [29,30]. This was also confirmed by a synchronous increase in the intensities of the electronic transitions of phosphorus and boron upon laser irradiation in our experiments.

### 3.2. Laser Power’s Impact on the Absorption Spectra

**Figure 5:**
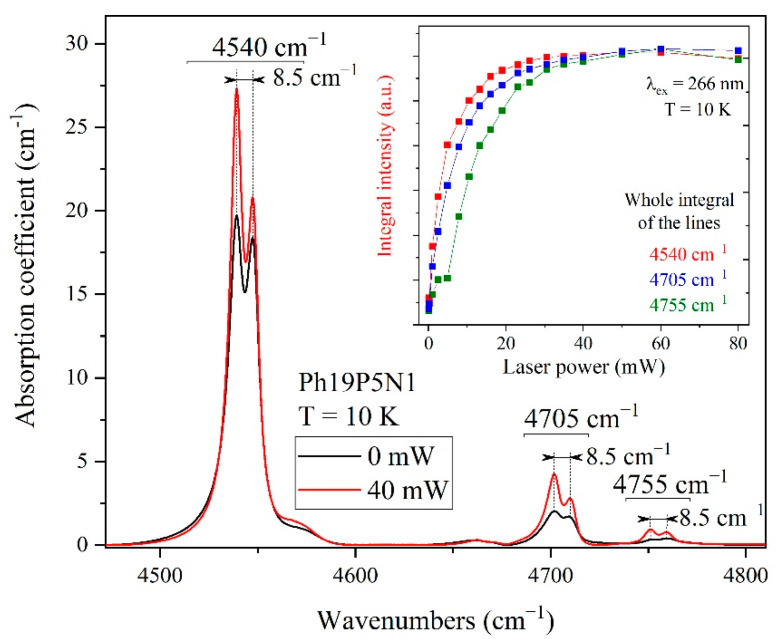
Absorption spectra of Ph19P5N1 sample in the region of phosphorus lines without (black) and with (red) laser irradiation with a wavelength of λ = 266 nm and a power of 40 mW. The inset shows the measured integral intensity of the doublet lines as a function of the laser radiation power.

The behavior of the absorption spectra of the Ph19P5N1 sample under UV laser irradiation (wavelength *λ* = 266 nm and a power 40 mW) is presented in more detail in Figure 5. An increase was registered in the intensities of all observed phosphorus lines, namely 4540 cm^−1^ (1*s* → 2p_±_), 4570 cm^−1^ (1*s* → 3p_0_), 4705 cm^−1^ (1*s* → 3p_±_), 4755 cm^−1^ (1*s* → 4p_±_). The inset in Figure 5 shows the dependence of the integral intensity of the lines at 4540, 4705 and 4755 cm^−1^ on the laser power (0–80 mW). It can be seen that the intensity of the lines reach saturation in the region of around 35 mW. The line width does not increase from 35 mW up to 80 mW, indicating that possible radiation-induced heating does not affect the results. The most intense phosphorus transition lines have a doublet structure, with a distance between the doublets of 8.5 cm^−1^, and are better resolved under laser irradiation, which is associated with the effect of a light-driven decrease in the line width. It was previously shown [9] that the doublet structure is associated with the dynamic Jahn–Teller effect, which splits the basic level 1*s*(T_2_) of phosphorus. The line narrowing under laser irradiation leads, in particular, to the observation of a biconvex line shape in the difference spectrum (Figure 4). It occurs due to the reduction in the Stark effect during the decompensation of ionized doping impurities. Similar observations of an increased intensity and narrowed lines were previously described when single-crystal silicon doped with electroactive impurities was irradiated with light [29].

**Figure 6:**
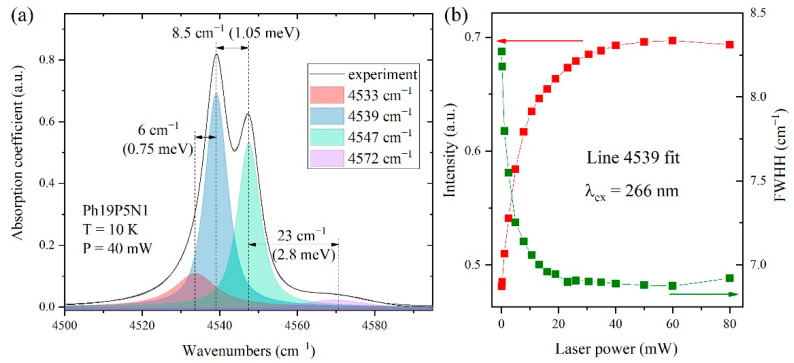
(**a**) Absorption band of phosphorus (4540 cm^−1^) decomposed into contours in the Ph19P5N1 sample under irradiation by UV laser with a wavelength of λ = 266 nm and a power of 40 mW; (**b**) characteristics of 4539 cm^−1^ individual contour line (intensity, width), depending on the laser radiation power.

The most intense absorption band of phosphorus, ~4540 cm^−1^, was decomposed into four components (Figure 6). In all cases, a decrease in the width of the component lines and, conversely, an increase in intensity is seen. All lines reach a plateau in the 35 mW region. In addition, the redistribution of the intensities of the doublet line at 4539 and 4547 cm^−1^ is observed (see also Figure 5), which is apparently due to the dynamic violation of the Boltzmann distribution under the action of laser radiation. We exclude the possibility of sample heating by laser radiation, since the linewidths do not change with the increase in the laser power. Furthermore, from the temperature dependence of the spectra without UV irradiation [9], it is known that the same distribution is observed at 80 K. Therefore, the used power densities of UV laser radiation could not heat the sample to this temperature.

### 3.3. Determination of the Total Concentration of Phosphorus Donors in Diamond

Based on our observations, we propose a method for the determination of the total concentration of electroactive impurities in a diamond. Our studies show that not all phosphorus impurities are involved in the optical absorption. The compensated impurities are ionized and do not absorb light in the IR spectral region. To obtain the total concentration, it is proposed to irradiate the sample cooled to cryogenic temperatures (10 K) by UV light. It is necessary to increase the UV radiation intensity until saturation of the decompensation effect is reached. The most convenient line for analysis is a doublet at 4540 cm^−1^. The integral intensity I_4540_ [cm^−2^] of this line under UV light irradiation is then calculated and the phosphorus concentration N_P_ [cm^−3^] is calculated using the following equation:

(1) $$N_{P}=I_{4540}\times A_{P}$$

where A_P_ [cm^−1^] is a correction factor. Using the correction factor A_P_ from work [24] (A_P_ = 2.7 × 10^14^ for 10 K measurements), we found both the total and net concentration of phosphorus, for both samples studied (see Table 1). As can be seen, a strong compensation ratio (68%) of donor phosphorus is observed for the sample Ph14N2, which is associated with the presence of a significant relative concentration of boron, along with a low concentration of phosphorus. For the Ph19P5N1 sample, a significantly lower compensation of donor phosphorus (13%) was observed. However, it can be seen from Table 1 that the concentration of acceptor impurities (boron) in these two diamond samples was at the same level, approximately 1.5 × 10^16^, which is consistent with the SIMS analysis (see Figure 1). Comparison of our SIMS and FTIR analyses shows that all phosphorus atoms in the studied samples occupied the substitution position, since the optically measured total concentration of the phosphorus under UV laser irradiation was in good agreement with the values measured using SIMS. In addition, apparently, the boron is the main decompensating impurity for phosphorus, since the number of compensated phosphorus donors was found to be equal to the concentration of boron acceptors, in accordance with the SIMS analysis (see Figure 1).

## 4. Conclusions

In this work, we have observed the effect of an increase in the intensity and a decrease in the half-width of the lines of electronic transitions of phosphorus impurities in diamond upon 266-nm laser irradiation. We have shown that the increased line intensity is associated with the decompensation of donors by the UV radiation near the diamond absorption edge, since the compensated donors and acceptors are ionized and do not participate in the IR absorption. Line narrowing occurs due to a decrease in the internal Stark effect, since ionized donors and acceptors in diamond are neutralized by laser action, and their effect on the electrical disorder in a diamond crystal disappears. We have demonstrated that the observed decompensation effect can be successfully used as a convenient method for the determination of both the total concentration and the compensation ratio of phosphorus centers in semiconducting diamond. The provided method is fully optical, nondestructive and does not require complex sample preparation, while being easy to perform and possessing high sensitivity (detection limit lower than 10^15^ cm^−3^).

## Figures and Tables

**Table 1:** Sample characteristics. Phosphorus donor concentrations (N_D_) from SIMS, the integral intensity of the spectral line without (I_4540_) and with (I_4540@UV_) laser irradiation, net (N_D_-N_A_) and total (N_D_tot_) phosphorus concentration from FTIR without and with UV irradiation, respectively, compensated phosphorus donors |N_D_-N_A_|-N_D_tot_ (N_D_comp_) and compensation ratio (k).

SAMPLE	N_D_ (cm^−3^)	I_4540_ (cm^−2^)	I_4540@UV_ (cm^−2^)	N_D_-N_A_ (cm^−3^)	N_D_tot_ (cm^−3^)	N_D_comp_ (cm^−3^)	k (%)
Ph14N2	2 × 10^16^	25 ± 5	78 ± 5	6.7 × 10^15^	2.1 × 10^16^	1.4 × 10^16^	68
Ph19P5N1	1.1 × 10^17^	353 ± 5	407 ± 5	0.95 × 10^17^	1.1 × 10^17^	1.5 × 10^16^	13
